# Effect of Sulfuric Acid Corrosion on Flotation Performance of Calcite by Changing Surface Roughness

**DOI:** 10.3390/molecules29051062

**Published:** 2024-02-29

**Authors:** Dingquan Xing, Ruofan Sun, Shuai Ma, Heping Wen, Zhongchi Wang, Jiushuai Deng

**Affiliations:** 1 Inner Mongolia Research Institute of CUMTB, Key Laboratory of Separation and Processing of Symbiotic-Associated Mineral Resources in Non-Ferrous Metal Industry, Engineering Technology Research Center for Comprehensive Utilization of Rare Earth—Rare Metal—Rare Scattered in Non-Ferrous Metal Industry, School of Chemical & Environmental Engineering, China University of Mining & Technology (Beijing), Beijing 100083, China; 15659121827@163.com (D.X.); srfyyds1996@126.com (R.S.); mashuai0929@163.com (S.M.); 2 Yuxi Dahongshan Mining Co., Ltd., Yuxi 653405, China; wehp1981@163.com; 3 School of Materials Science and Engineering, Inner Mongolia University of Technology, Hohhot 010051, China; wzc020731@163.com

**Keywords:** calcite, surface roughness, flotation, sulfuric acid corrosion, AFM

## Abstract

Surface roughness is a crucial factor that affects the flotation performance of minerals. In this study, the effect of sulfuric acid corrosion on the surface roughness of calcite flotation was investigated through microflotation tests, scanning electron microscopy (SEM–EDS), atomic force microscopy (AFM), Fourier transform infrared (FT-IR) spectroscopy, and contact angle analysis. Microflotation test results show that sulfuric acid treatment has a serious negative effect on the floatability of calcite. When the sulfuric acid dosage was 4 mL (3 mol/L), the flotation recovery of calcite was reduced to less than 19%. SEM–EDS and AFM results verified that the sulfuric acid treatment significantly changed the surface morphology of calcite, reduced the average surface roughness and surface area, and reduced the amount of active Ca^2+^ sites on the calcite surface. As characterized by FT-IR and contact angle analyses, the sulfuric acid treatment enhanced the hydrophilicity of the calcite surface and reduced the amount of sodium oleate adsorbed on the calcite surface. Consequently, sulfuric acid corrosion can reduce the average surface roughness of calcite and have a serious negative effect on the flotation performance of calcite.

## 1. Introduction

Calcite (CaCO_3_) is one of the most widely distributed carbonate minerals on Earth [1], and often coexists with apatite (Ca_3_(PO_4_)_2_), fluorite (CaF_2_), and scheelite (CaWO_4_) [2,3]. In mineral processing, calcite is often regarded as a gangue mineral and must, therefore, be separated from other useful minerals [4,5,6]. Foam flotation is an effective method for separating calcite from other calcium-containing useful minerals [7,8,9]. In the flotation tank, hydrophobic particles connected to the bubbles during flotation, were pulled upward by the rising bubbles, and then created a froth on top of the suspension. Hydrophilic particles, however, were unable to be captured by the bubbles and stayed at the bottom of the suspension [10,11]. Therefore, the surface properties of minerals significantly influence the flotation separation effect. Nevertheless, calcite exhibits similar surface properties to calcium-containing minerals (e.g., scheelite, fluorite, and apatite), resulting in difficulties in separating these minerals by flotation [12,13,14,15]. Therefore, the effective separation of these minerals is a challenge in the field of flotation of calcium-containing minerals [16].

Research on calcite flotation has primarily focused on the development and utilization of high-efficiency depressants [9]. To date, three categories have been established for calcite depressants: inorganic, organic, and mixed [17]. Inorganic depressants are mainly water glass and its modified products (e.g., acidified water glass) [12,18,19,20]. Its mechanism of action mainly involves the water glass forms Si(OH)_4_ or SiO_2_(OH)_2_^2−^ adsorption on the calcite surface in the pulp, which hinders the adsorption of the collector [17]. Starch, dextrin, and sodium carboxymethylcellulose are the most commonly used organic depressants [21,22,23]. For the depressive effect of starch, starch molecules are adsorbed on the calcite surface to form a hydrophilic film, which enhances the hydrophilicity of the mineral surface [24]. Combination depressants often contain a combination of three or more different depressants, creating a synergistic effect and exhibiting more potent selective depression. Common depressant combinations include denim and starch [23,25], starch and dextrin [26], sodium lignosulfonate and sodium humate [27], Cu^2+^ and starch [28], and sodium humic acid and water glass [17].

The inhibition effect of the aforementioned depressants reduces the surface hydrophobicity of calcite minerals by selective adsorption on the mineral surface and enhances the difference between the surface hydrophobicity and that of the minerals to be floated [9]. However, during industrial processes, owing to the complex pulp environment and several interfering ions, the activity and selectivity of flotation agents are affected, resulting in the flotation separation of minerals, making it difficult to achieve the desired effect [10].

The surface roughness of the mineral is also an important factor affecting its flotation performance [29]. Surface roughness, which is typically created by the processing technique and other variables, is the narrow spacing and small peak and valley roughness of a treated surface [30]. The hydration film on the mineral surface is affected by the surface roughness of the mineral (e.g., stability and deformation) and hinders the adhesion between the particles and bubbles in the floating process [31]. Over the past few decades, the impact of varying surface roughness on the behavior of mineral flotation has been extensively studied.

Ahmed et al. [32] proved that the grinding method has an effect on the surface roughness of minerals, including larger specific surface areas and greater amounts of microstructural defects, resulted in an increased adsorption capacity of the reagent, more stable flotation foam, and faster flotation kinetics. Li et al. [33] demonstrated that an increase in the microscale roughness of a mineral surface can improve the flotation of minerals. However, other studies provide contrasting results. Hi (Cyilmaz) et al. [34] demonstrate that barite grains with higher surface smoothness exhibit better floatability under the condition of using A-845 (Cytec) succinamate as a surfactant. Ulusoy et al. [35] found that when the surface roughness of talc, quartz, and barite particles decreased, the surface contact angle and floatability of these minerals increased. In summary, changing the surface roughness can change the flotation effect of minerals. However, the effect of surface roughness on the flotation performance of different minerals is different, and no uniform conclusion is possible.

Deng et al. [36] found that when calcite reacts with sulfuric acid, the mineral surface dissolves to form a CaSO_4_ layer, which may affect its surface roughness. In addition, the authors studied the separation of fluorite and calcite [5], and found that sulfuric acid pretreatment would affect the floatability of calcite. The sulfuric acid pretreatment was only used as a means, and the detailed mechanism of the effect of sulfuric acid on the floatability of calcite was not deeply investigated, which is not conducive to revealing the mechanism affecting minerals separation. Therefore, in the current study, the surface roughness of calcite was modified using sulfuric acid, and the differences in mineral flotation behavior under different degrees of corrosion were explored. Experimental investigations to determine the role of surface roughness in calcite flotation include microflotation experiments, scanning electron microscopy (SEM–EDS), Fourier transform infrared (FT-IR) spectroscopy, atomic force microscopy (AFM), and contact angle measurements. The findings of this study will provide a fundamental understanding of the role of surface roughness in calcite flotation.

## 2. Results and Discussion

### 2.1. Microflotation Results

**Figure 1:**
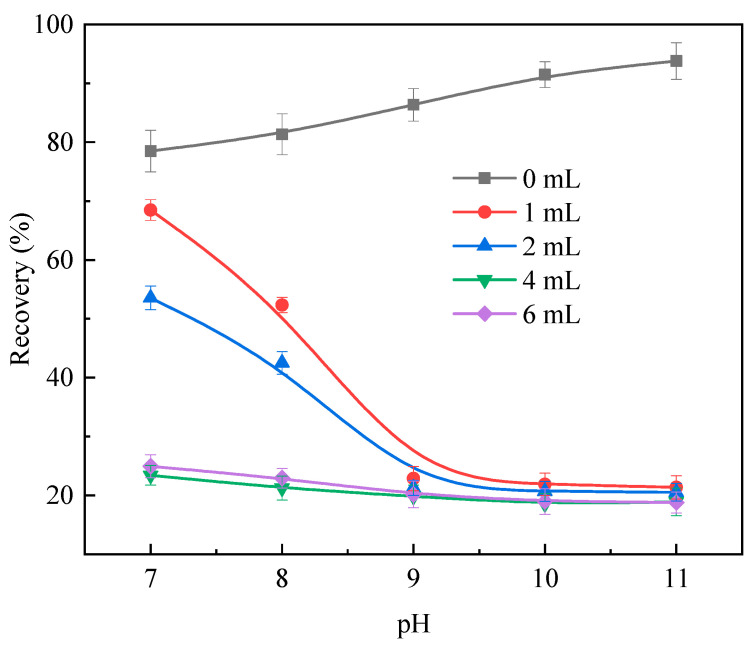
Effect of H_2_SO_4_ dosage on flotation performance of calcite particles.

Microflotation experiments were performed to investigate the effect of the sulfuric acid corrosion treatment on the floatability of the calcite particles. The flotation results are shown in Figure 1; NaOL dosage was 60 mg/L, slurry pH range was 7–11.

As presented in Figure 1, over the entire experimental pH range, calcite exhibited excellent floatability, and with the continuous increase in pulp pH, the flotation recovery slowly increased. When the pH value of the pulp reached 11, the recovery of calcite reached the highest value of 93.79%. However, after treatment with sulfuric acid, the overall calcite recovery decreased significantly. When the dosage of sulfuric acid was 2 mL or less, the recovery of calcite first decreased with an increase in the pulp pH, then stabilized, and finally decreased to approximately 20%. After the dosage of sulfuric acid was increased to 4 mL, the change in the pH of the slurry had little effect on the recovery of calcite, which remained stable at approximately 19%, and a further increase in the acid dosage had little impact on the recovery. These results indicate that a certain dosage of sulfuric acid could effectively weaken the floatability of calcite.

### 2.2. Surface Morphology Characterization

**Figure 2:**
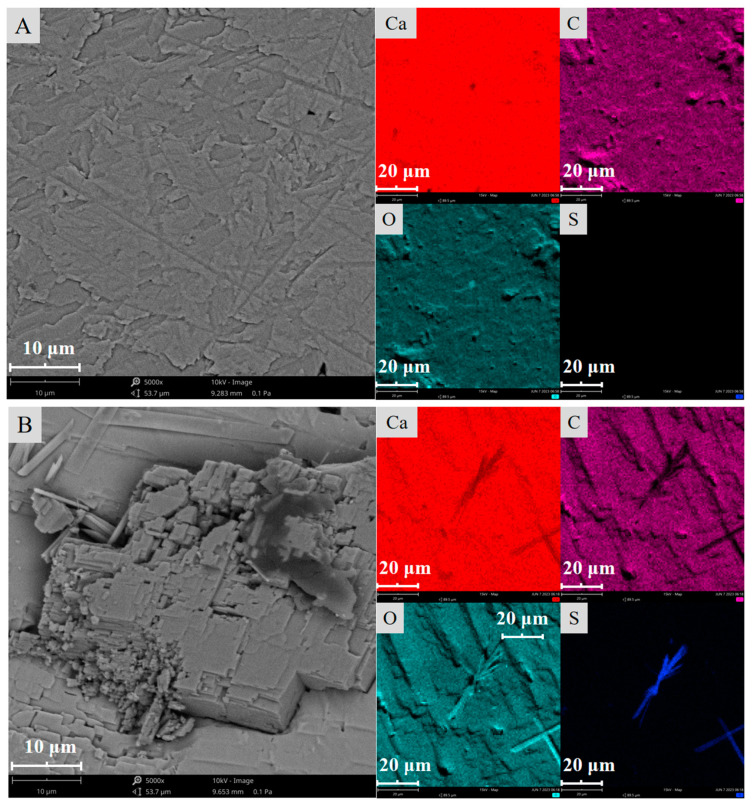

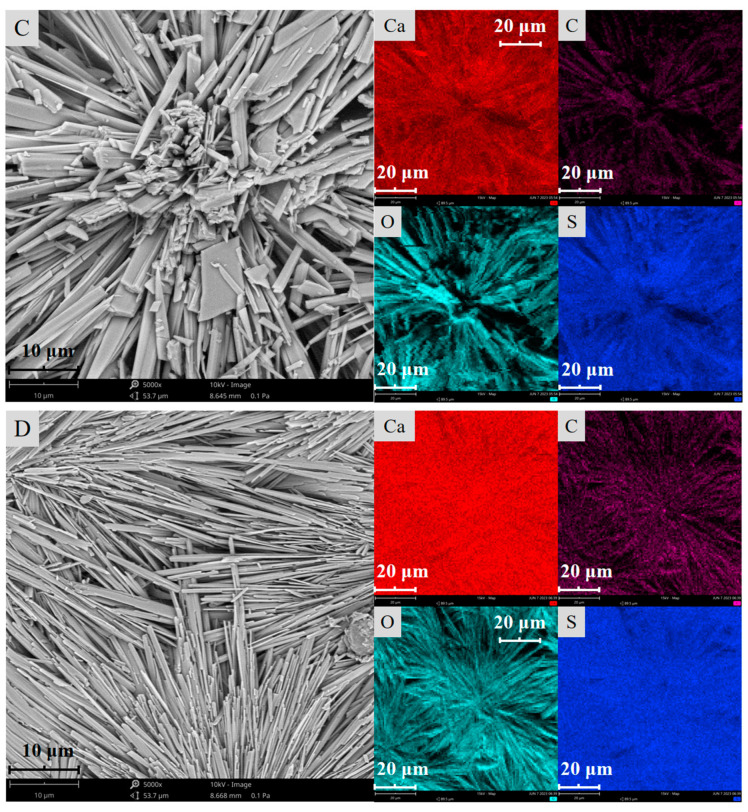
SEM images of calcite samples ((**A**) H_2_SO_4_, 0 mL; (**B**) H_2_SO_4_, 1 mL; (**C**) H_2_SO_4_, 2 mL; (**D**) H_2_SO_4_, 4 mL; H_2_SO_4_ concentration, 3 mol/L).

The surface morphology and chemical composition of the calcite absence and presence of sulfuric acid treatment were measured using SEM–EDS, and the results are presented in Figure 2 and Table 1.

As illustrated in Figure 2 and Table 1, before sulfuric acid treatment, the surface morphology of calcite was relatively flat, and the mineral surface was composed of Ca, O, and C. After adding 1 mL of sulfuric acid, a small number of crystal clusters appeared on the calcite surface, and 0.37% of the S element was added to the surface. Simultaneously, the C element decreased by 0.45 percentage points, which was speculated to be a small amount of CaCO_3_ converted into CaSO_4_ on the calcite surface [36]. When the sulfuric acid dosage was increased to 2 mL, the surface of calcite was nearly completely covered by the generated CaSO_4_ crystal clusters, S content increased to 13.43%, C content further decreased by 7.17 percentage points, and Ca content also decreased by 14.31 percentage points. When the amount of sulfuric acid was increased to 4 mL, the surface morphology of the calcite hardly changed and the weight percentage of the surface elements remained unchanged.

These results demonstrate that after the addition of sulfuric acid, the surface morphology of calcite changes significantly because of the generated CaSO_4_ crystal clusters, which might be the main reason for the decreased floatability of calcite.

### 2.3. Surface Roughness Characterization

**Figure 3:**
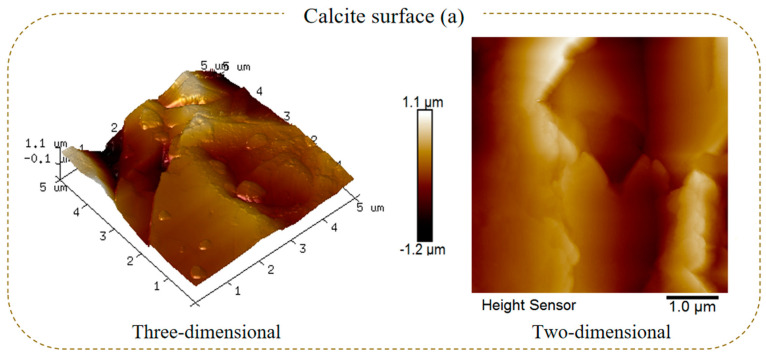

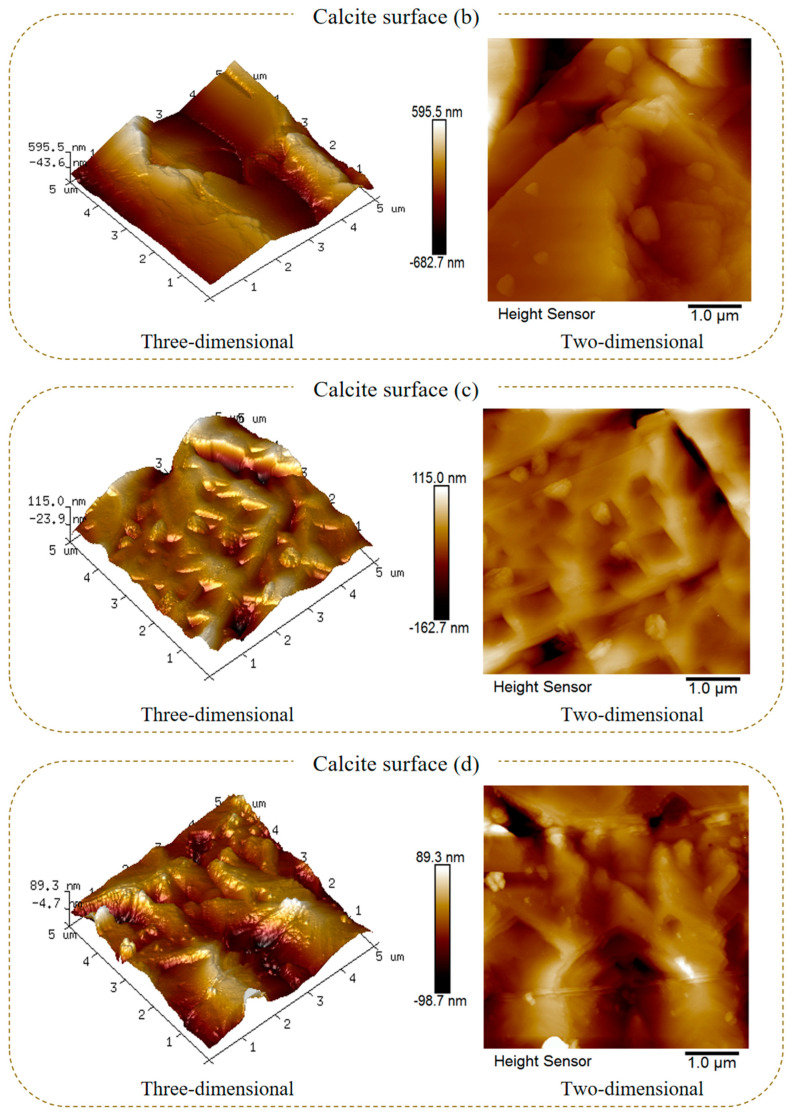
AFM images of calcite particles ((**a**) H_2_SO_4_, 0 mL; (**b**) H_2_SO_4_, 1 mL; (**c**) H_2_SO_4_, 2 mL; (**d**) H_2_SO_4_, 4 mL; H_2_SO_4_ concentration, 3 mol/L).

**Figure 4:**
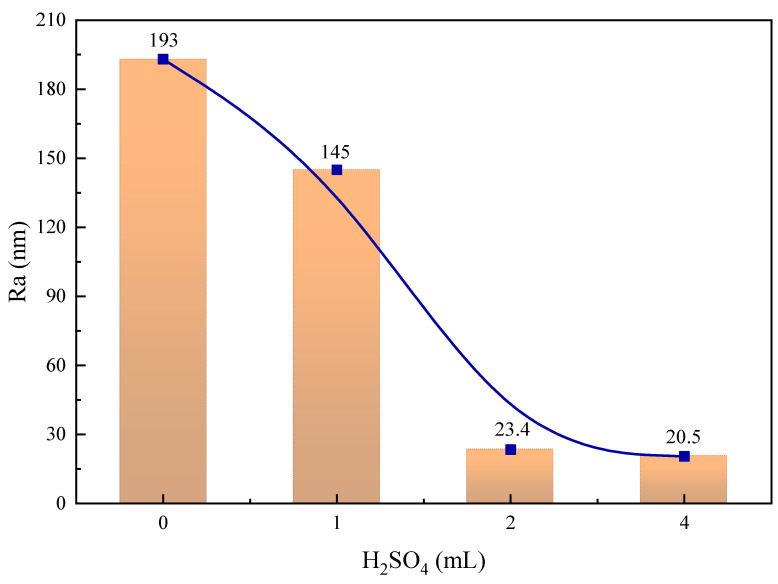
Average surface roughness of calcite particles determined by AFM method.

AFM was used to further study the changes in the surface morphology and roughness after the reaction of calcite with sulfuric acid. Figure 3 shows the representative two-dimensional and three-dimensional AFM images of 5.0 × 5.0 μm^2^ of calcite particles under different acid dosage conditions, and the related average surface roughness is simultaneously given in Figure 4 by statistical calculation for five AFM images.

In Figure 3, the color bar shows the height of the calcite surface, where the darkest and lightest colors indicate the lower and higher parts of the calcite surface, respectively [31]. From the two-dimensional analysis, a significant difference in the distribution of lighter and darker colors on the calcite particle surfaces was observed under the four conditions. The color difference on the raw calcite surface was the largest, indicating that the depth of the mineral surface was greater. As the amount of sulfuric acid increased, the color difference gradually decreased, indicating that the depth of the mineral surface also decreased. Three-dimensional analysis shows that the raw calcite surface was covered with large, wide peaks and deep valleys. Similarly, as the dosage of sulfuric acid increased, the wide peaks and deep valleys on the mineral surface gradually disappeared and became gentle with smooth hilly slopes (Figure 3d). Combined with the SEM–EDS results, it can be speculated that the cause of this phenomenon is that sulfuric acid reacted with calcite to dissolve the wide peaks of the surface protrusions, and a CaSO_4_ product was formed to fill the deep valleys.

**Figure 5:**
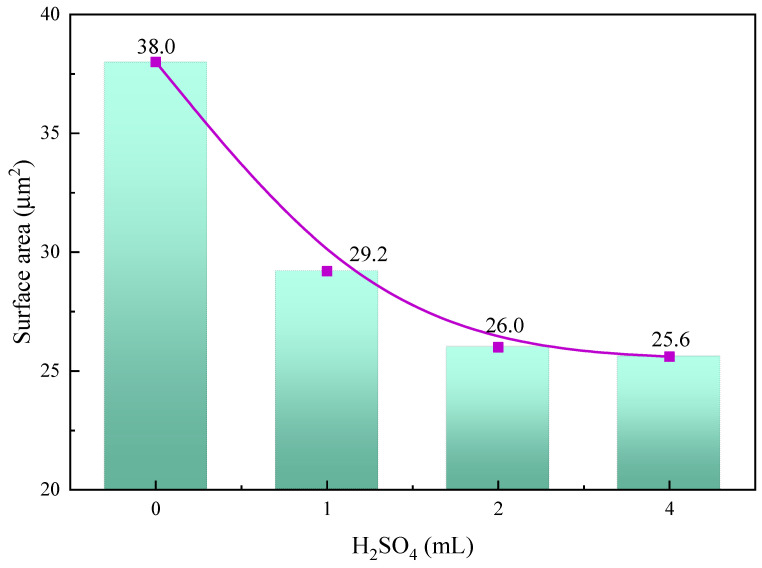
Surface area of calcite particles determined by AFM method.

As shown in Figure 4, the average surface roughness of the calcite particles decreased significantly with increasing sulfuric acid consumption. The average surface roughness of raw calcite was 193 nm, and after using 4 mL of sulfuric acid, the average surface roughness of calcite decreased to 20.5 nm. This indicates that sulfuric acid corrosion can significantly lower the average surface roughness of calcite. In addition, Figure 5 illustrates the change in calcite particle surface area. The mineral surface area also decreased significantly with the increase in acid dosage, from 38 μm^2^ of the raw ore to 25.6 μm^2^ of 4 mL of acid consumption.

Therefore, from the aforementioned results, acid corrosion reduced the wide peaks and deep valleys on the surface of calcite, average surface roughness, surface area, and a number of collector action sites, resulting in a decrease in the floatability of calcite, which is consistent with the results of the microflotation test.

### 2.4. Surface Composition Analysis

**Figure 6:**
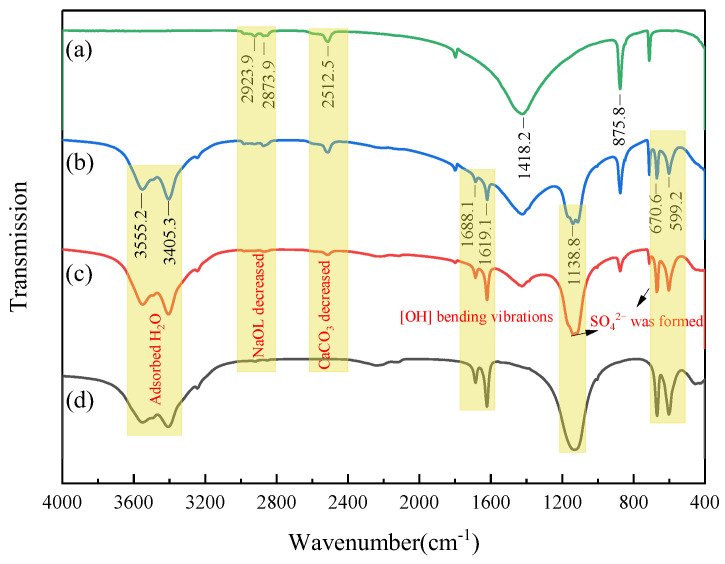
FT-IR spectra of calcite samples with and without different reagents ((**a**) calcite + NaOL, (**b**) calcite + H_2_SO_4_ (1 mL) + NaOL, (**c**) calcite + H_2_SO_4_ (2 mL) + NaOL, (**d**) calcite + H_2_SO_4_ (4 mL) + NaOL, NaOL (30 mg/L)); H_2_SO_4_ concentration, 3 mol/L.

In order to further study the influence mechanism of calcite flotation behavior in sulfuric acid corrosion treatment, FT-IR spectrometry was applied to detect the new species formed on the calcite surfaces, and to explore the effect on NaOL adsorption. The FT-IR analysis results of the calcite treatment with different reagents are shown in Figure 6.

In Figure 6a, characteristic peaks of calcite can be observed at wavelengths 2512.5, 1418.2, and 875.8 cm^−1^ [37,38], and the peaks at 2923.9 and 2873.9 cm^−1^ are the symmetric and asymmetric stretching vibration peaks, respectively, of –CH_2_– in NaOL [39,40], indicating that NaOL has been adsorbed on the calcite surface. After treatment with different dosages of H_2_SO_4_, the FT-IR spectra of calcite changed significantly. As illustrated in Figure 6b–d, three new peaks appear at 599.2, 670.6, and 1138.8 cm^−1^, which can be attributed to the bending vibration of SO_4_^2−^ [41], indicating that CaSO_4_ forms on the calcite surface at this time.

Observing the strength of the calcite surface peak, it can be seen that with the gradual increases in acid dosage, the characteristic peak of CaCO_3_ on the calcite surface gradually weakened, while the characteristic peak of CaSO_4_ strengthened, indicating that the CaCO_3_ component on the calcite surface was transformed into CaSO_4_, which was consistent with the SEM–EDS results. More importantly, the NaOL peak also gradually decreased with increasing acid dosage, and when the acid dosage was 4 mL, the characteristic peak of NaOL was almost absent on the calcite surface. The reason for this phenomenon may be that after treatment with sulfuric acid, the specific surface area and Ca^2+^ active sites of calcite decreased, resulting in reduced NaOL adsorption. Additionally, water absorption peaks appeared on the surface of calcite treated with sulfuric acid, with the absorption peaks at 3555.2 and 3405.3 cm^−1^ arising from the O–H stretching vibration and the peaks at 1619.1 and 1688.1 cm^−1^ resulting from O–H bending vibrations [28,42], indicating that the surface of calcite became hydrophilic after the H_2_SO_4_ treatment.

### 2.5. Surface Wettability Measurements

Variations in the contact angle reflect differences in the hydrophobicity of mineral surfaces before and after reagent treatment [43]. The larger the contact angle, the more hydrophobic the surface of the mineral, and vice versa, the more hydrophilic of the mineral surface [44]. Therefore, in this section, contact angle measurements were used to study the change in surface hydrophobicity of calcite under different agent conditions.

**Figure 7:**
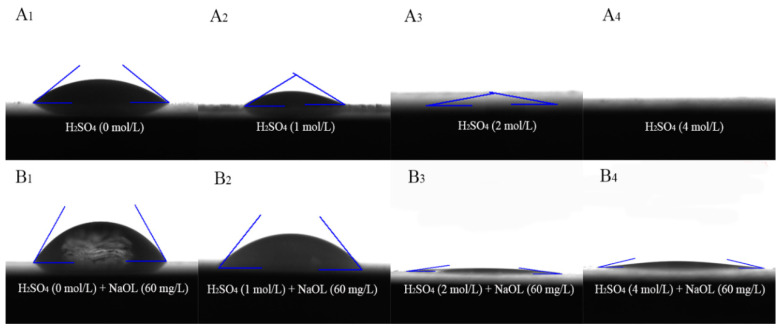
Contact angle images of calcite samples: A. calcite + H_2_SO_4_ (A_1_ (0 mL), A_2_ (1 mL), A_3_ (2 mL), A_4_ (4 mL)); B. calcite + H_2_SO_4_ + NaOL (B_1_ (0 mL + 60 mg/L), B_2_ (1 mL + 60 mg/L), B_3_ (2 mL + 60 mg/L), B_4_ (4 mL + 60 mg/L)); H_2_SO_4_ concentration, 3 mol/L.

The results are presented in Figure 7 and Table 2. The contact angle of raw calcite was 37.14°, which signifies high hydrophilicity and poor floatability [45], consistent with previous reports [46]. After treatment with sulfuric acid, the contact angle of the calcite decreased rapidly with increasing amounts of sulfuric acid. With an acid dosage of 2 mol/L, when the droplets contacted the calcite surface, they immediately diffused and infiltrated the mineral, resulting in a completely hydrophilic calcite surface. According to the results of the SEM–EDS and AFM measurements, when sulfuric acid reacted with calcite, the surface changed from flat CaCO_3_ to CaSO_4_ crystal clusters, resulting in a decrease in the specific surface area and average roughness of calcite. The generated CaSO_4_ crystal clusters loosely cover the surface of calcite, resulting in a significant enhancement in the hydrophilicity of the calcite surface.

After treatment with NaOL, the contact angle of the raw calcite increased to 58.93°, indicating that the adsorption of NaOL significantly enhanced the hydrophobicity of the mineral surface. However, the contact angle of calcite treated with sulfuric acid still decreased rapidly but was higher than that of calcite untreated with NaOL, indicating that a small amount of NaOL was adsorbed on the mineral surface, which was consistent with the FT-IR analysis results. Therefore, sulfuric acid treatment significantly reduced the hydrophobicity of the calcite surface. In addition, the use of NaOL exerted a very weak effect on the hydrophobicity of calcite, resulting in the floatability of calcite being maintained at an extremely low level, which is consistent with the results of the microflotation test.

### 2.6. Possible Mechanism

Combining the results of the microflotation test and surface roughness detection, it can be concluded that the average surface roughness of calcite has a significant impact on its flotation behavior. With an increase in the sulfuric acid dosage, the average surface roughness and surface area of calcite exhibited a downward trend; correspondingly, the floatability of calcite was also significantly weakened. The SEM–EDS, FT-IR, and contact angle results further demonstrate that a higher average surface roughness and surface area could provide more active sites, and the sulfuric acid treatment converted CaCO_3_ on the surface of calcite into CaSO_4_ with a lower average surface roughness and smaller surface area. This considerably enhanced the hydrophilicity of the calcite surface and significantly interfered with the adsorption of the collector NaOL. Therefore, it can be concluded that sulfuric acid treatment can convert the undulating CaCO_3_ on the calcite surface into loose CaSO_4_ crystal clusters, significantly reducing the average roughness and surface area of calcite. This results in the reduction in Ca active sites on the calcite surface, finally causing the enhancement of mineral surface hydrophilicity, weakening its interaction with NaOL, and negatively affecting the floatability of calcite.

## 3. Materials and Methods

### 3.1. Materials and Reagents

**Figure 8:**
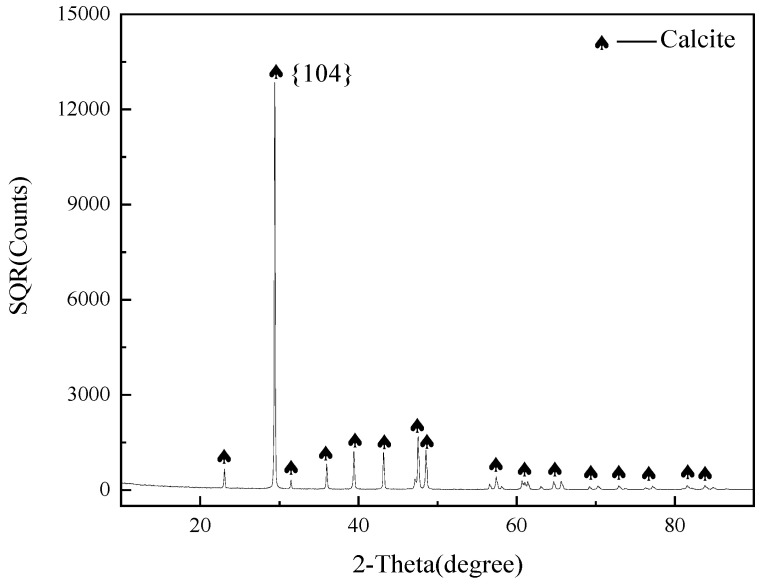
XRD patterns of pure calcite sample.

The calcite pure mineral samples were bought from Yunnan Province, China. A hammer was applied to break up high-purity lump minerals to produce nugget samples. The sheet samples with regular surfaces were prepared for SEM analysis. The remaining samples were further ground using an agate mortar and screened to produce a product with a particle size of −45 m for X-ray diffraction (XRD) and FT-IR analysis, and samples with a particle size of 45–105 m for microflotation testing. The XRD patterns of pure calcite samples are illustrated in Figure 8. The results reveal that these samples are highly pure, matching with the standards of pure minerals.

In this experiment, 2 mol/L sodium hydroxide (NaOH) was used as slurry pH adjuster, and 3 and 1 mol/L sulfuric acid (H_2_SO_4_) were used as the reaction reagent and slurry pH adjuster, respectively, while the collector was sodium oleate (NaOL). Table 3 includes a list of the agents employed in this investigation. Ultrapure water (18 MΩ/cm) was used throughout the experiment.

### 3.2. Microflotation Experiments

Microflotation experiments were carried out in an laboratory RK/FGC5-35 flotation machine, and the stirring speed and airflow was 1600 rpm and 15 cm^3^/min, respectively. In each test, 2 g of pure calcite sample and 60 mL of deionized water were added to the flotation cell and stirred for 1 min. Next, 3 mol/L H_2_SO_4_ (0–6 mL) (if needed) was added to the flotation cell and stirred for 5 min. Then, dilute NaOH and H_2_SO_4_ solutions were added to adjust the pH of the slurry. After that, collector NaOL (60 mg/L) was added to the cells for 3 min. After opening the aeration valve, the foam was manually scraped for 3 min to collect the froth products. Finally, the froth products and tailings were filtered, dried, and weighed to calculate the recovery, and each test was repeated thrice.

### 3.3. AFM Measurements

Both submicroscopic and microscopic surface topographies of the calcite particles were studied using AFM under specific test conditions. The surface roughness of the calcite was quantified using the arithmetic average (*R_a_*) and root mean square (*R_q_*). The related parameters *R_a_* and *R_q_* are as follows [31]:

(1) $$R_{q}=\sqrt{\frac{\sum {\left(Z_{i}-Z_{\mathrm{ave}}\right)}^{2}}{N}},\;$$

(2) $$R_{a}=\frac{1}{L_{x}L_{y}}\int_{0}^{L_{y}}\int_{0}^{L_{x}}\left|f(x,y)\right|\mathrm{dxdy},\;$$

where *N* is the number of points within the given area, *Z_i_* represents the current *Z* value, *Z_ave_* is the average of the *Z* values (the *Z*-axis represents topographical height features), *f*(*x*,*y*) describes the surface’s relationship to the center plane, and *L_x_* and *L_y_* represent the surface’s various dimensions.

In this investigation, peak force tapping mode on a MultiMode 8 AFM (Bruker Germany, Karlsruhe, Germany) was used to collect AFM images of the calcite particle surfaces. First, glue was used to attach calcite particles on a glass slide. The surface of the calcite was imaged using five different particles that were chosen at random. The corresponding averages and standard deviations were then determined for both the *R_q_* and *R_a_* values that had been reported. The pictures shown here depict the usual topography of calcite surfaces.

### 3.4. SEM–EDS Analysis

The surface morphology and elements of the calcite were examined using scanning electron microscopy (SEM) and energy dispersive spectroscopy (EDS) (Regulus SU8230, Hitachi, Tokyo, Japan). The resin A glue and B glue were mixed with a mass ratio of 3:1, stirred for 5 min to obtain transparent mixed resin glue, the flake calcite sample (5 × 5 × 3 mm) was placed in a silica gel mold with a diameter of 1.0 cm, and then the resin glue was poured and allowed to stand for 24 h to obtain the glued calcite sample. Then, 200, 800, 1500, 3000, and 5000 mesh silicon carbide sandpaper were used to polish the glued calcite sample surface. The samples were then processed with 3 mol/L sulfuric acid, reacting for 5 min. After that, the mineral samples were coated with gold using a sputter coater after being dried in a vacuum oven at 40 °C. Finally, the processed samples were examined using a Phenom (Ambler, PA, USA) ProX equipment at a 10 kV accelerating voltage.

### 3.5. FT-IR Measurement

A Spectrum One FT-IR spectrometer (Nexus 670; Thermo Nicolet, Vacaville, CA, USA) was used to perform the FT-IR spectroscopy. In a beaker, a sample (2.0 g) with a size fraction of −5 μm was mixed with 60 mL of deionized water. Next, the mineral samples were treated under the same conditions as microflotation, and then the samples were filtered and dried in a vacuum oven at 40 °C. Finally, 1 mg samples were mixed with 100 mg KBr, and then tested using the pressure plate method. The spectra were taken between 400 and 4000 cm^−1^.

### 3.6. Contact Angle Measurements

DSA255 goniometer (DSA25S, KRŰSS, Hamburg, Germany) was used to determine the contact angles of calcite samples. The lamp samples were polished with silicon carbide sandpaper of 400, 600, 1200, 1500, 2000, 3000, and 5000 mesh, and then cleaned three times with hydrochloric acid and ultrapure water. After being vacuum dried at 40 °C, the calcite samples were processed with H_2_SO_4_ and NaOL; the treatment conditions were consistent with those for the microflotation. Using the sessile drop method to measure the contact angle of the calcite sample, the ADVANCE software (German KRŰSS, Version 1.9.2.2) automatically calculated data based on the profile of the surface droplets. To generate averages and confirm the accuracy of experimental results, each sample was measured in triplicate.

## 4. Conclusions

In this study, the effects of sulfuric acid treatment on the flotation performance of calcite were systematically investigated by varying its surface roughness. The mechanism was demonstrated using microflotation tests, SEM–EDS, AFM, FT-IR spectroscopy, and contact angle measurements. The following conclusions were drawn from the experiments:

(1) The results of the microflotation experiments suggest that sulfuric acid treatment can significantly reduce the floatability of calcite, and the flotation recovery of calcite was stable at approximately 19% when the acid dosage was greater than 2 mL;
(2) The SEM and AFM results reveal that after the sulfuric acid reaction of calcite, its surface changes from stepped CaCO_3_ to crystalline cluster CaSO_4_, which significantly reduces the average surface roughness and surface area of calcite and the active sites Ca^2+^;
(3) The FT-IR results and contact angle measurements reveal that the surface of calcite treated with sulfuric acid exhibits strong hydrophilicity. Moreover, the acid treatment reduces the adsorption of NaOL, which makes it difficult for NaOL to enhance the hydrophobicity of the calcite surface.

## Figures and Tables

**Table 1:** Surface element weight percentage of calcite samples (H_2_SO_4_ concentration, 3 mol/L).

H_2_SO_4_ Dosage (mL)	Surface Element Weight Percentage (%)			
	Ca	C	O	S
0	34.47	15.13	50.40	-
1	33.76	14.68	51.09	0.37
2	20.16	7.51	58.90	13.43
4	20.88	7.79	56.35	14.98

**Table 2:** Contact angle of calcite samples under different treatment conditions: A. calcite + H_2_SO_4_ (A_1_ (0 mL), A_2_ (1 mL), A_3_ (2 mL), A_4_ (4 mL)); B. calcite + H_2_SO_4_ + NaOL (B_1_ (0 mL + 60 mg/L), B_2_ (1 mL + 60 mg/L), B_3_ (2 mL + 60 mg/L), B_4_ (4 mL + 60 mg/L)); H_2_SO_4_ concentration, 3 mol/L.

Treatment Conditions	A_1_	A_2_	A_3_	A_3_
Contact angle (θ_A_)	37.14	29.47	5.16	None
**Treatment conditions**	**B_1_**	**B_2_**	**B_3_**	**B_4_**
Contact angle (θ_A_)	58.93	49.26	7.71	12.72

**Table 3:** Details of the agent.

Chemical	Conc. %	Supplier	Role
H_2_SO_4_	98.0	Sinopharm Chemical	pH adjuster
NaOH	96.0	Shiyi Chemical Reagent	pH adjuster
NaOL	99.0	Macleans Biochemical	Collector

## Data Availability

The data presented in this study are available in article.
